# Loss and Recovery Potential of Marine Habitats: An Experimental Study of Factors Maintaining Resilience in Subtidal Algal Forests at the Adriatic Sea

**DOI:** 10.1371/journal.pone.0010791

**Published:** 2010-05-24

**Authors:** Shimrit Perkol-Finkel, Laura Airoldi

**Affiliations:** Dipartimento di Biologia Evoluzionistica Sperimentale and Centro Interdipartimentale di Ricerca per le Scienze Ambientali, University of Bologna, Ravenna, Italy; Dalhousie University, Canada

## Abstract

**Background:**

Predicting and abating the loss of natural habitats present a huge challenge in science, conservation and management. Algal forests are globally threatened by loss and severe recruitment failure, but our understanding of resilience in these systems and its potential disruption by anthropogenic factors lags well behind other habitats. We tested hypotheses regarding triggers for decline and recovery potential in subtidal forests of canopy-forming algae of the genus *Cystoseira*.

**Methodology/Principal Findings:**

By using a combination of historical data, and quantitative *in situ* observations of natural recruitment patterns we suggest that recent declines of forests along the coasts of the north Adriatic Sea were triggered by increasing cumulative impacts of natural- and human-induced habitat instability along with several extreme storm events. Clearing and transplantation experiments subsequently demonstrated that at such advanced stages of ecosystem degradation, increased substratum stability would be essential but not sufficient to reverse the loss, and that for recovery to occur removal of the new dominant space occupiers (i.e., opportunistic species including turf algae and mussels) would be required. Lack of surrounding adult canopies did not seem to impair the potential for assisted recovery, suggesting that in these systems recovery could be actively enhanced even following severe depletions.

**Conclusions/Significance:**

We demonstrate that sudden habitat loss can be facilitated by long term changes in the biotic and abiotic conditions in the system, that erode the ability of natural ecosystems to absorb and recover from multiple stressors of natural and human origin. Moreover, we demonstrate that the mere restoration of environmental conditions preceding a loss, if possible, may be insufficient for ecosystem restoration, and is scarcely cost-effective. We conclude that the loss of complex marine habitats in human-dominated landscapes could be mitigated with appropriate consideration and management of incremental habitat changes and of attributes facilitating system recovery.

## Introduction

Throughout history, humans have severely modified or exploited to complete loss >70% of natural habitats in the habitable portion of the planet [1], and further loss is expected to occur at a rate of 0.5–1.5% of wild nature per year [2]. Although habitat loss is well recognized as a key threat to global biodiversity and human livelihood, understanding and abating the effects of habitat loss and fragmentation still represents a huge challenge in science, conservation and management [3], [4], [5]. An increasing number of studies have demonstrated that sudden losses do not only depend on proximate triggers (sudden disturbances or perturbations) but also on a gradual history of changes in biotic and biophysical conditions (climate, pollution, habitat quality, resource availability), that erode the ability of natural ecosystems to absorb and recover from multiple stressors of natural and human origin [6], [7], [8]. Further, once resilience of natural systems is eroded, the mere restoration of environmental conditions preceding the loss, if possible, might not suffice for recovering the system [9]. In fact, highly degraded systems may require a recovery pathway which differs from the original trajectory of change [10], [11]. The failure of some populations and ecosystems to naturally recover from human stressors has stimulated much interest in resilience building and restoration management [12], [13]. Successful ecosystem recovery is still likely for many systems [14], but it requires extensive knowledge of drivers of loss that must be regulated and of mechanisms facilitating recovery, much of which is currently lacking for many natural systems.

Strong declines of marine forests of kelps, fucoids and other complex, canopy-forming macroalgae have occurred over the past decades around the world [15], [16], [17]. This is concerning because algal forests play a key role in coastal primary production, nutrient cycling and disturbance regulation, and create complex habitats that facilitate abundant plant and animal communities and rival corals for sheer diversity of species [15]. Lost canopies tend to be replaced by species with lower structural complexity, such as turf-forming, filamentous or other ephemeral seaweeds, mussels, or urchin “barrens” (e.g., [18], [19], [20]) leading to a widespread perception that the coastal marine seascapes are becoming increasingly flatter. There is uncertainty about the underlying drivers of the loss of marine canopies. Local anthropogenic stressors such as direct degradation or destruction of habitat, eutrophication, sedimentation, and overfishing, as well as episodic disturbances from outbreaks of urchins, storms, disease and direct harvesting are often evident as proximate triggers of these declines [18], [19], [21], [22]. However factors controlling the subsequent recovery are notoriously more difficult to identify [23], [24], [25]. Thus, while there are examples of recovery of canopies from outbreak of urchins (e.g., [26], [27]), or severe acute disturbance events e.g. El Nino-Southern Oscillations, [28], [29], recovery potential for lost algal forests seems to be limited in many regions of the world, particularly where forests become permanently replaced by turfs, sediments, or mussels [17], [18], [30]. The causes for such severe recovery failure are unclear, and could include a combination of modified habitat and biotic conditions, demographic factors (e.g. reduced fertility) and/or low dispersal potential [24], [31], [32]. While new strategies for restoration and conservation are constantly evolving, taking into account the resilience of the system in light of cumulative impacts, incorporating threshold models and feedback mechanisms [9], [10], [33], their application to kelp forest dynamics is still lacking.

We tested hypotheses regarding triggers for decline as well as recovery potential of degraded marine algal forests by using a combination of historical data, quantitative *in situ* observations of natural recruitment patterns and experimental work. Our study area consisted of the degraded algal forest of the canopy-forming alga *Cystoseira barbata* C. Agardh (Fucales: Sargassaceae), along the urbanized coasts of the north Adriatic Sea. In this region, as well as in the whole Mediterranean Sea, *Cystoseira* forests have suffered particularly widespread and persistent loss. Along the Albéres coasts (south France) only 5 out of 14 species of Fucales (*Cystoseira* and *Sargassum* spp.) documented as abundant in 1912 were still present in 2003, with the genus *Sargassum* entirely lost [19]. Similar trends are occurring along the coasts of the Tyrrhenian [18] and Ligurian seas [20] and southern Adriatic sea [34]. Remaining fragments of algal forests are under continued threat, so much so that 6 Mediterranean species of *Cystoseira* are listed in the Bern Convention and the Mediterranean Action Plan, adopted within the framework of the Barcelona Convention. This plan identifies the conservation of *Cystoseira* belts as a priority, but so far the overall benefits of these protection measures have been low.

We used both historical data and quantitative *in situ* observations of natural patterns of distribution and recruitment for documenting the degradation and loss of canopy habitats in the study region over the past 70 years, and their replacement by opportunistic species. Our observations have lead us to the hypothesis that recent loss of forests along the urbanized coasts of the north Adriatic Sea were triggered by increasing cumulative impacts of natural- and human-induced habitat instability and severe recruitment failure. By using clearing experiments we subsequently tested whether greater substratum stability would be sufficient to reverse the loss, or whether for recovery to occur removal of the new dominant space occupiers (i.e., opportunistic species including turf algae and mussels) would also be required. We finally used transplantation experiments to identify the conditions that could facilitate natural recovery and guide possible restoration efforts of canopy-forming algae.

## Materials and Methods

### Study area and species

**Figure 1:**
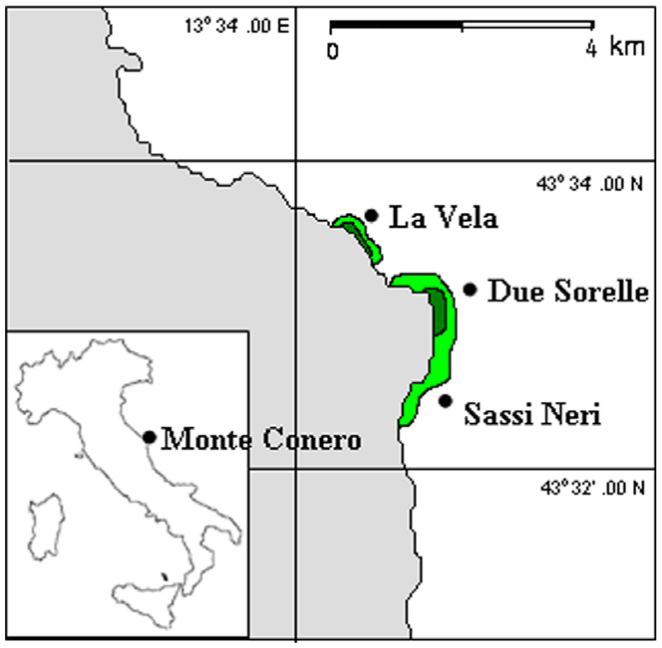
Map of the study region: superimposed is the known distribution of *Cystoseira* canopies until 2001 (light green, from: [40]) and since 2006 (dark green, data from the present work).

The study took place around the Monte Conero promontory (Fig. 1), on the border between the North and Central Adriatic Italian coast (43°33′N, 13°37′E). This 8 km long sea cliff represents the seaward limit of a regional natural park and one of the few natural rocky outcrops along the otherwise extensively urbanized sandy coastline of the Italian Adriatic. The rocky reefs are composed mainly of marls and limestones and extend to about 8 m in depth. Major human pressures in the area are represented by tourist activities and infrastructures.

The degraded canopy forest still present in the area is one of the very few remaining in the central-northern Italian Adriatic coast. It is highly isolated, being surrounded both northwards and southwards by sandy habitats. Nowadays the forest is mainly composed by the species *C. barbata* that can be found in varying densities at depths of about 2 to 5 m. *C. compressa* is the only other species sparsely present, particularly at shallower depths. As typical for the genus, canopies show a seasonal pattern characterized by growth of dense thalli in spring-summer, up to a height of 50 cm and fall of phylloids and branchelets in autumn-winter [35]. *C. barbata*, as most members of the genus, is considered monoecious, and according to our observations [36], it develops mature conceptacles and releases gametes from end of March until late June.

### Past and current canopy distribution

We reviewed available published records of historical distribution of *Cystoseira* forests in the area from both the scientific and grey literature. Available published information covered the period between 1941 and 2001 and consisted mainly of floristic lists of species (presence/absence), a map from 2000 illustrating the distribution of *Cystoseira* spp. dominated habitats along the Monte Conero promontory and an impact study of the effects of nourishments in 2001 (see results). We integrated this data with direct personal interviews conducted with local port authorities, dive club operators, and civil protection forces to gain a deeper understanding of the abundance of these habitats prior to 2000. Questions mainly focused on the historic location and abundance of *Cystoseira* canopies in the area, as well as their impact on tourism and boating activities. Information was then crossed with information published in the literature and overall collected information was in agreement with published data. More recent information originated from occasional field observations of the occurrence of dense canopy fronds carried out by LA between 1999 and 2001 during other studies in the area.

In 2006 our group started to work systematically on canopy forests. At that time a preliminary survey was done at about 15 locations around the promontory noting the presence or absence of canopy forests (F. Colosio, LA et al, unpublished data). Only two sparsely forested areas could be found in the region located at the sites called hereon “Due Sorelle”, and “La Vela”, about 2 km apart (Fig. 1). In July 2008 we extensively mapped these areas using line transects (50 m long, four transects per site). Along each transect we recorded changes in dominant benthic components (i.e. dense *Cystoseira* canopies vs. alternative habitats such as concentrations of *Cystoseira* recruits, mosaic of various benthic components intermixed with sporadic fronds of *Cystoseira*, patches of anemones and turf, mussel beds, sand patches etc.). This allowed us to quantify the current degree of fragmentation of the forested habitat, the size of the remaining patches of *Cystoseira* and the abundance of other alternative benthic habitats. At each site, we also estimated the average cover, density and height of thalli within the few patches of *Cystoseira* using 5 replicated quadrats of 50×50 cm. Given the very small size of canopy patches (see Results) we sampled a single quadrat per patch.

### Substratum characteristics and wave regime

Since our observations pointed towards a possible role of substratum instability in shaping the local *Cystoseira* forests, we collected information on whether significant changes in substratum characteristics had occurred in the past decades. Moreover, as during severe storms the waves scour the rocky bedrocks exerting severe mechanical action, we also collected historical wave data to analyze whether there had been an increase in the frequency or intensity of extreme wave events in the area. Yearly frequencies of significant wave heights during 1999–2004 were obtained from APAT through the RON (Rete Ondametrica Nazionale) network from a wave recording buoy located just offshore our study sites (43.49.47 N; 13.42.52 E). Comparable data from 2005 to 2010 were not available due to repeated damages to the measuring buoy. We also derived an estimate for the level of substratum instability in the area under the effects of severe storm through monitoring all the marked substrata used for the various experiments described in the following sections of “Material and methods”. For these experiments >30 boulders ranging from <1 m^3^ to >10 m^3^ were permanently marked at the two study sites using epoxy putty (Subcoat S., Veneziani), a two component marine glue with a clay-like consistency which hardens when mixed and exposed to water, either for use as experimental plots or as reference markers for the study sites. Marks were regularly monitored since July 2008 until February 2010, allowing us to estimate the proportion of boulders that were overturned or moved during storms.

### Natural patterns of recruitment

In July 2008, just after the reproductive season, we sampled the distribution of *C. barbata* recruits in order to quantify natural recovery in the system and to test how this was influenced by the dominant habitats that replace the lost canopies. At both study sites, we extensively searched for recruits from the surface down to 6 m in depth below canopies of *Cystoseira* and within mussel beds, stands of simpler seaweeds, or mosaics of these two habitats. In all these habitats, recruitment of *Cystoseira* was virtually nil (see Results), and no further sampling was carried out.

At both sites we found, however, occasional patches of extremely dense recruits of *Cystoseira* on numerous shallow (<3 m in depth) boulders (see Results). We monitored the fate of these recruits by permanently marking some of these boulders with epoxy putty. Because the size of the boulders was variable, which could affect their stability as well as other properties related to substratum size, we tested whether boulder size could influence post recruitment survival of *Cystoseira* by marking 3 large (>4 m^3^) and 3 small (<1 m^3^) boulders at La Vela and Due Sorelle. Percent cover of recruits was estimated at the centre of each boulder using 0.3×0.3 m frames subdivided into 25 small (6×6 cm) sub-quadrats, each representing 4% of the frames' surface area [37]. We also estimated densities of recruits in 5 sub-quadrats at random. Such measurements were time consuming, due to the difficulty of separating individual juvenile thalli at such high densities, therefore we limited them to recruits on large boulders, which we hypothesized had the greatest chance of survival. Because of difficulties in measuring the height of dense thalli in the field, at each site we also removed all juveniles from one random 6×6 cm plot on each of 3 non-marked boulders covered with juveniles, for subsequent measurements in the laboratory. For this destructive sampling, we used different boulders every time. Sampling was repeated in July, September, and October 2008 and in February 2009. Differences in percent cover of juveniles between boulders of different sizes were tested by using a 2-way ANOVA on data from October 2008 (as on the next sampling time in February 2009 cover of juveniles was virtually 0), including factors Size of boulders and Site.

### Clearing experiments

We used clearing experiments to analyze possible causes for the severe recruitment failure of *C. barbata* in the area and to quantify the recovery potential of the system. Specifically we tested whether: 1) *Cystoseira* has the potential to recruit onto substrata other that the unstable one where recruits are naturally found; 2) recruitment potential of *Cystoseira* differs between shallow areas where scattered adult canopies still occur and deeper areas at the border of its natural distribution where canopies are virtually lacking; 3) recruitment is inhibited by the presence of alternative habitats such as mussels and simpler seaweeds; 4) recovery potential differs throughout the reproductive season of *Cystoseira*. The experiment was initiated in March 2009, which coincided with the beginning of the reproductive window for *C. barbata*. At each of the two sites, La Vela and Due Sorelle, 10 plots of 0.3×0.3 m were marked by using epoxy putty at depths of about 3 m, where dense adult canopies still occur. Five plots at random were scraped clean by hammer, chisel and scraper, until bare rock was completely exposed, while the remaining half were left as un-manipulated controls. The same types of plots, interspersed at random in the experimental area, were produced again in late May, at the end of the reproductive period for *Cystoseira*. At this time, 10 additional plots (5 cleared and 5 controls) were also set at each site at a depth of ca. 5 m where canopies are virtually absent.

Recruitment of *Cystoseira* was quantified by counting all recruits visible to the naked eye (i.e., >0.5 cm) that developed in the experimental plots. Recruits were sampled in June and September 2009, and in February 2010 (at this last time rough sea conditions prevented us from completing the sampling of the deepest plots). Recruitment and survival of recruits were compared using ANOVAs. We ran two analyses. One tested for the effects of Treatment, Time of clearing and Site, and used data from shallow sites only, from the last sampling time in February 2010. The second, tested for the effect of Treatment, Depth and Site, and used data from plots cleared in May and sampled during September 2009 (due to missing data for deep plots in February 2010).

### Transplantation experiments

We used transplantation experiments to indentify the conditions that could facilitate the recovery and guide possible restoration efforts of canopy algae. Specifically we tested if juvenile survival is enhanced: 1) by a greater stability of the bottom substrata, 2) by the presence of a nearby surrounding adult canopy, and 3) on horizontal rather than vertical substrata. We tested the latter hypothesis because human interventions that modify habitat characteristics in increasingly urbanized marine areas (e.g. by coastal armoring) tend to increase the relative abundance of vertical compared to horizontal substrata [38], [39], and our observations suggest that these could be less favorable to algal canopies. At each site, small sized boulders (ca. 0.1×0.1 m) densely covered with recruits of *C. babata* were collected from zones of low survival probability (i.e., small, shallow boulders), broken into small fragments holding 1–2 individuals, and transplanted in plots of 5 individuals using epoxy putty onto more stable bedrocks (boulders >10 m^3^ in size or eroded rocky platforms). Here recruits were set: 1) horizontally with adults, where juveniles were transplanted onto a horizontal stable surface surrounded by adult fronds, 2) horizontally without adults, where juveniles were transplanted onto a horizontal stable surface without surrounding adult fronds, and 3) vertically, where juveniles were transplanted on vertical stable surfaces which generally are devoid of adult fronds. Controls comprised recruits manipulated and transplanted back to their original location. Transplantations were carried out during July 2008, when juveniles had reached a manageable size of ca. 5 cm. Four replicated plots of each treatment were prepared at each site. Survival and growth of juveniles were estimated by counting and measuring the height of thalli in each plot in September and October 2008 and in February 2009.

Since at Due Sorelle no plots survived a storm in December 2008 (see Results), we compared differences in survival rate of transplanted juveniles using data from October 2008, by using running a 2-way ANOVA including factors Site and Treatment. For treatments in La Vela we ran an additional 1-way ANOVA on the last data collected in February 2009.

## Results

### Past and current canopy distribution

According to past published data on the floristic composition and distribution of algal populations at Monte Conero from 1941 until 2000 ([40] and reference therein) in the 1940's the canopy forests of Fucoid algae in the area were much more diverse than today, including as many as 7 species of *Cystoseira*, and one species of *Sargassum*. Only 4 species of *Cystoseira* still occurred in the 1960's and since the 1990's only two species of *Cystoseira* (*C. barbata and C. compressa*) have been found. Until 2000 canopies of *Cystoseira* were documented to cover an area approximately 4 km long. Personal observations by LA confirm that this distribution of canopies was broadly maintained until late 2001. Interviews with personnel and authorities from the local recreational marina indicated that at some locations (such as the location named Sassi Neri), fronds were so large and abundant as to occasionally present obstacles to boats and fishermen, and that fronds washed up to shore occasionally disturbed recreational beach users.

In early 2006 our field observations reported the presence of canopy forests only at two localities named “La Vela” and “Due Sorelle” where fronds of *Cystoseir*a covered areas of about 300 m parallel to the shoreline at a depth range of 2–4 m, while we could no longer find any canopies along the long coastal stretch of Sassi Neri (this novel distribution is superimposed on the past map of distribution in Fig. 1), Therefore we concluded that canopies of *Cystoseira* were lost rapidly sometimes between 2002 and 2005. Canopies have not recovered so far, despite regular supply of propagules (see next section “Natural patterns of recruitment”).

**Figure 2:**
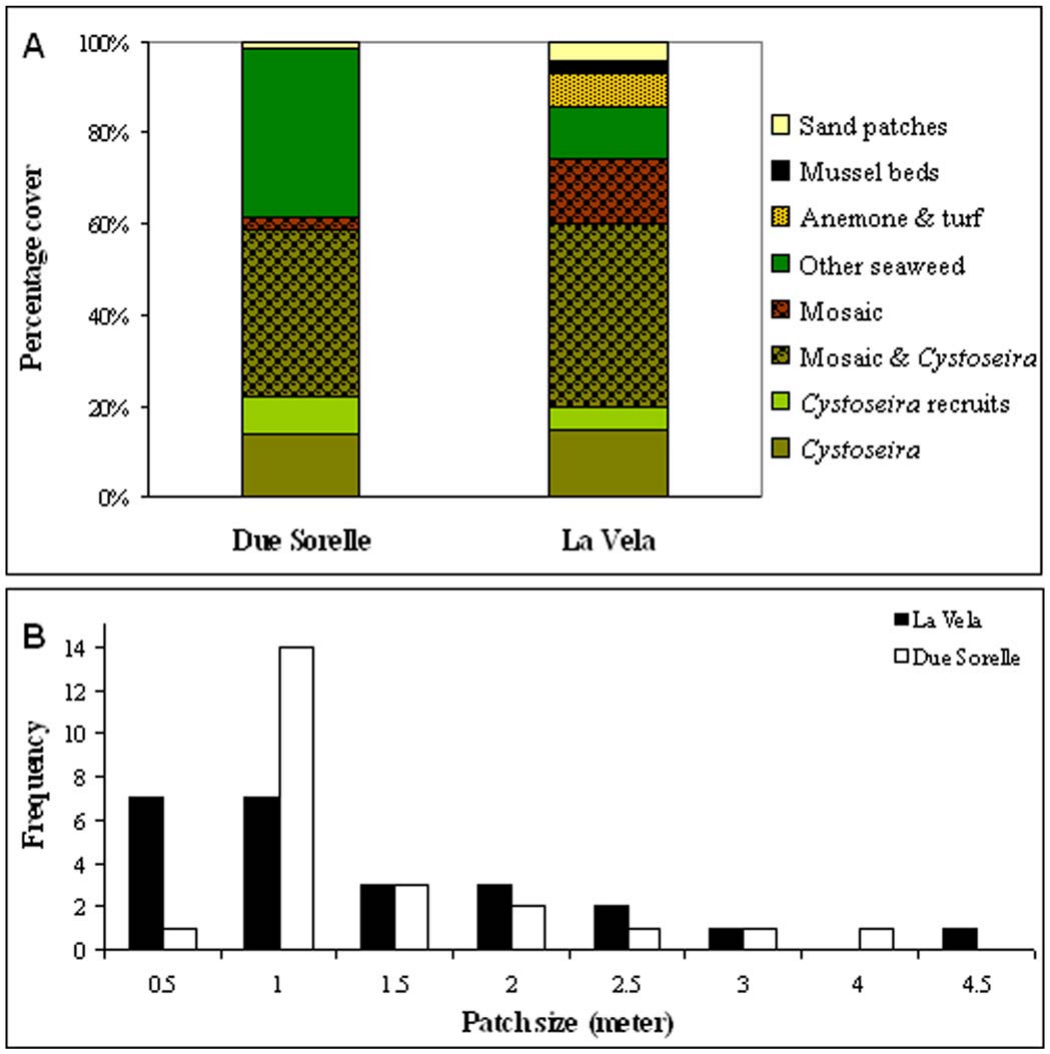
Characterization of the *Cystoseira* canopies at the study site: **A**. Average percentages of bedrock occupied by canopies of *Cystoseira* (mainly *C. barbata*, occasional fronds of *C. compressa*) or other relevant biogenic habitats; and **B**. Size frequency distribution of patchy canopies of *Cystoseira* at each of the two study sites. Data were obtained from four 50 m line transects conducted at each site in July 2008. For details about the habitats see results.

Sampling in 2008 revealed that the remaining stands of *Cystoseira* at La Vela and Due Sorelle were very fragmented, hardly covering >15% of the sampled bedrocks between 2 and 4 m (Fig. 2a), while only sparse fronds of *Cystoseira* occurred deeper than that, frequently mixed with mosaics of other habitats. The high level of fragmentation of *Cystoseira* canopy was clearly evident from the patch size analysis (Fig. 2b) with most patches smaller than 1.5 m, and never larger than 4.5 m. Density of *Cystoseira* fronds within the most frequent patch size averaged 16.61±3.27 (SE) fronds (per 50×50 cm quadrat) and frond length averaged 24.27±1.24 cm.

At both sites, much of the bedrocks were occupied by various biogenic habitats of lower structural complexity (Fig. 2a), mainly comprising stands of simpler seaweeds such as *Chondracanthus acicularis*, *Gelidium latifolium*, *Gigartina* spp. and *Ulva* spp, beds of *Mytilus galloprovincialis*, thin filamentous algal turfs often accompanied with *Anemonia viridis*, or mixed heterogeneous mosaics of these components.

### Substratum characteristics and wave regime

In the study area, the rocky bottom is naturally physically unstable due to the tendency of the steep arenaceous cliffs of Monte Conero to erode originating boulders and cobbles. The trend has been historically enhanced by human activities related to rock mining occurring in the 1930's–1940's, and more recently to extensive nourishments conducted between 1997 and 2001 at a number of beaches along the promontory by placement of gravel and quarry stones, ranging in size from few centimeters up to 1 m, at the base of the cliffs. Monitoring carried out just after the nourishments in 2001 [41] showed that in the areas affected by the interventions the cover of *Cystoseira* immediately decreased by 20% and cover of cobbles increased by 20%.

**Figure 3:**
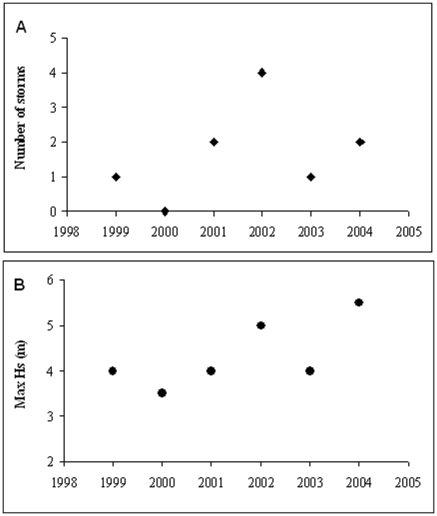
Wave regime at the study region: **A**. Number of sea storms (significant wave height H_s_ >3.5 m) and **B**. maximal wave height recorded each year from 1999–2004 by a waver recording buoy located just offshore the study sites (43.49.47 N; 13.42.52 E). Data are from APAT through the RON (Rete Ondametrica Nazionale) network (courtesy Dr. G. Nardone).

In the study region, severe storms were particularly frequent in 2002 (Fig. 3a), and 2002 and 2004 were exceptional for severity of storms (Fig. 3b), with the oceanographic buoy offshore Monte Conero registering significant wave height H_s_ of >5.5 m in September 2004 [42]. Nonetheless, during our experiments, a severe storm hit our study sites in December 2008. On this occasion, a buoy located offshore Cesenatico (44.2155°N 12.4766°E), about 130 km north of our study area, recorded a significant wave height of 3.2 m (data from ARPA, Servizio IroMeteoClima). During the storm all marked experimental substrata <4 m^3^ were displaced and/or overturned. At the locality of Due Sorelle, which was hit particularly strong, displacement and overturning also affected larger boulders >10 m^3^, while at the locality La Vela substrata >4 m^3^ were relatively stable.

### Natural patterns of recruitment

**Figure 4:**
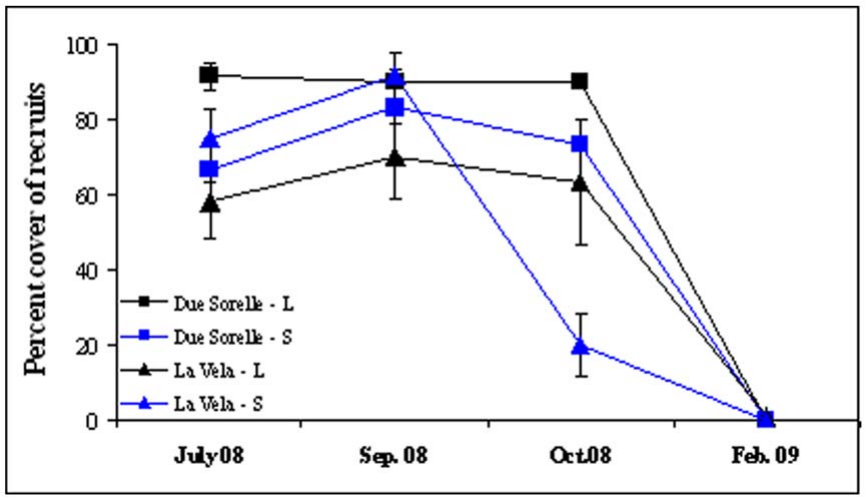
Natural changes in cover of recruits: Average percent cover (±1SE, n = 3 plots of 30×30 cm) of *C. barbata* recruits on large (>4 m^3^, L-black) vs. small (>1 m^3^, S-blue) marked boulders from July 2008 to February 2009 at Due Sorelle (squares) and La Vela (triangles).

**Figure 5:**
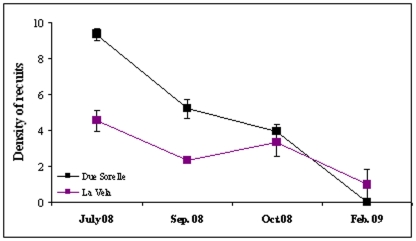
Natural changes in density of recruits: Average densities (±1 SE, n = 15, 5 sub-quadrats of 6×6 cm from each of 3 plots of 30×30 cm) of *C. barbata* recruits of on large (>4 m^3^) marked boulders from July 2008 to February 2009 at Due Sorelle (black) and La Vela (purple).

We found virtually no recruits neither underneath canopies of *Cystoseira* nor in any other of the dominant habitats comprising mussel beds, stands of simpler seaweeds, or mixed mosaics of the two. In all these habitats recruits, if any, occurred only very sporadically (<1 recruits per 2.5 m^2^). However, at both sites we found occasional patches of extremely dense recruits of *Cystoseira*, reaching up to >90% cover (Fig. 4) and densities as high as 16 juveniles per 6×6 cm sub-quadrat (Fig. 5). These patches of recruits were mainly confined to shallow boulders otherwise generally covered by thin filamentous turfs or other small ephemeral algae. On average, cover and density of recruits were highest at Due Sorelle (significant effect of factor Site for cover, *F*_1,8_ = 15.97, P<0.01), and based on our transects we estimated they accounted for 5 to 8% of the surveyed area at La Vela and Due Sorelle, respectively (Fig. 2a).

Initial average size of recruits in June 2008 was 4.67±0.18 (SE) cm. Recruits persisted on both large and small marked boulders throughout the summer, but by October 2008, after the first moderate storms, their cover (Fig. 4) started to decrease, particularly on the smallest boulders (significant effect of factor Size, *F*_1,8_ = 9.74, P<0.05). At both sites, cover and density of recruits sharply decreased leading to complete disappearance following the storm in December 2008 (Figs. 4 and 5), when all marked boulders, regardless of their size, were either severely displaced or overturned. The average size of the few surviving recruits we could still find in the study area in February 2009 was 8.27±0.35 cm.

### Clearing experiments

**Figure 6:**
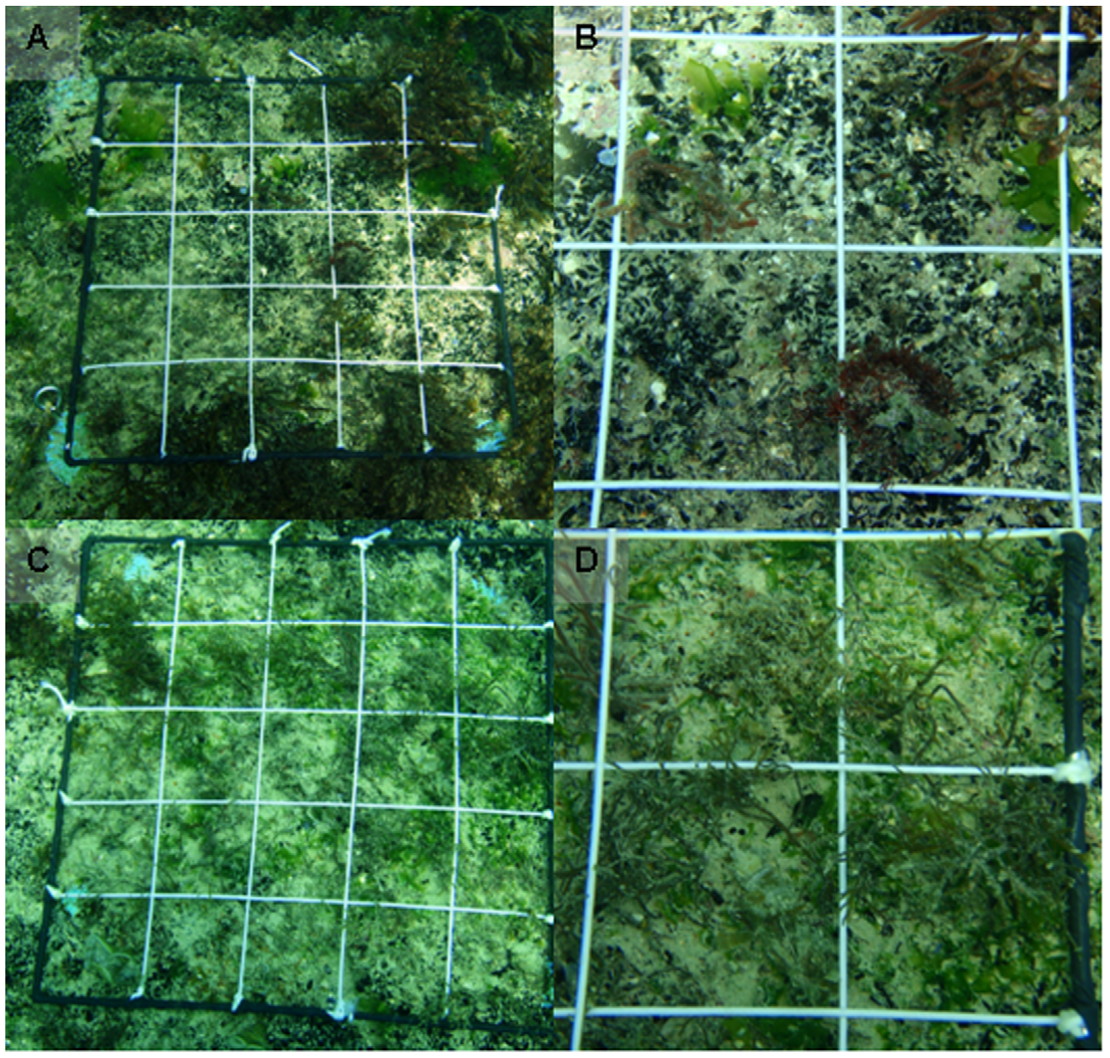
Photos of the Clearing Experiment: **A**. un-manipulated (control) plot showing typical cover of stable habitats composed of various (non-*Cystoseira*) seaweeds, mussel beds and turf. **B**. Representative close-up of control plot with no *Cystoseira* recruits. **C**. Cleared plot dense ca. 3 m old *C*. *barbata* recruits. **D**. Close-up of ca. 3 m old *C*. *barbata* recruits in cleared plot.

**Figure 7:**
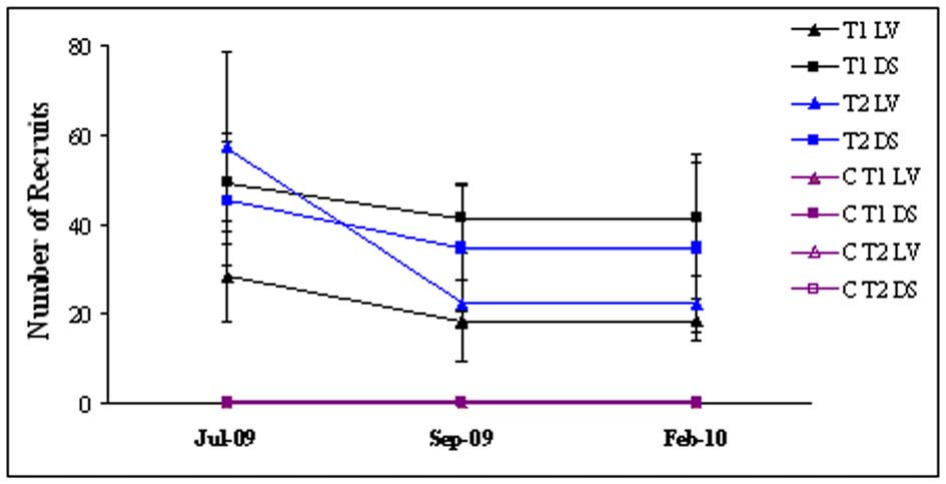
Variations in recruitment patterns throughout the reproductive season: Average densities (±1SE, n = 5 plots of 30×30 cm) of *C. barbata* recruits in plots cleared in March 2009 (beginning of reproductive season, T1 in black), vs. plots cleared in May 2009 (end of the reproductive season, T2-in blue), at La Vela (LV-triangles) and Due Sorelle (DS-squares) from July 2009 to February 2010. There were un-manipulated control plots (C-in purple) at both clearing times and sites, but all had virtually no recruits and thus the points in the graph overlap. All the plots were located in shallow areas where canopies of *C. barbata* are present.

**Figure 8:**
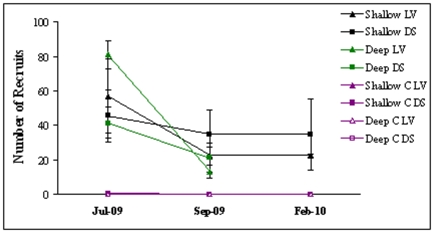
Variations in recruitment patterns between shallow and deep areas of the habitat: Average densities (±1SE, n = 5 plots of 30×30 cm) of *C. barbata* recruits in experimental plots at La Vela (LV-triangles) and Due Sorelle (DS-squares) from July 2009 to February 2010. Plots were either cleared in shallow areas (2.5 m depth, black), were canopies of *C. barbata* are present, or cleared in deeper areas (5 m depth, green), where no canopies are normally found. There were un-manipulated control plots (C-purple) at both depth and sites, but all had virtually no recruits and thus the points in the graph overlap.

Clearing experiments demonstrated that *Cystoseira* has a high recruitment potential, but this is limited by competition with the new space occupiers. On control un-manipulated plots, which were covered by mixed mosaics of mussels and algae of low structural complexity (Fig. 6a), there was virtually no recruitment of *Cystoseira* (Fig. 6b). Conversely, any cleared plot was rapidly covered with recruits of *Cystoseira* (Fig. 6c–d), which reached densities as high as 133 juveniles per 30×30 cm (Fig. 7). The plots used in this experiment were all located on stable eroded rocky platforms which were not damaged by the severe winter storms. After an initial decrease in density, the remaining recruits in the experimental plots have survived the winter and grew to young adults. There were no differences related to the time of clearing (Fig. 7, *F*_1,32_ = 0.02, P>0.05) indicating that the potential for recruitment is high throughout the reproductive season of *Cystoseira*. Similarly, there were no differences in recruitment between plots cleared at shallower areas with presence of surrounding adult canopies and plots cleared in deeper areas without adult canopies (Fig. 8, no effect of Depth *F*_1,32_ = 1.63, P>0.05), indicating that the lack of canopies at deeper sites is not related to dispersal limitation.

### Transplantation experiments

**Figure 9:**
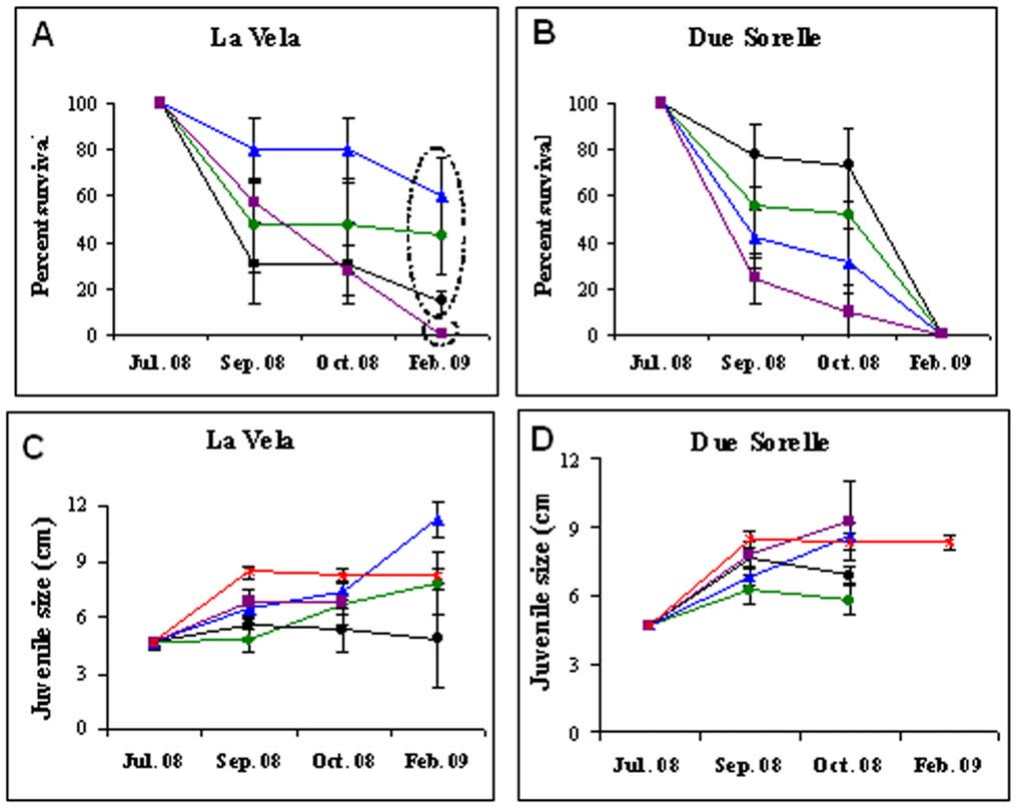
Results from the transplantation experiments: **A**.**–B**. Average percent **Survival** and **C**.**–D**. average **Size** of *C. barbata* juveniles transplanted at each study site from July 2008 to February 2009. Treatments were transplanted to: horizontal substrata surrounded by adults (green), horizontal substrata without adults (black), vertical substrata without adults (blue) and original unstable control substrata (purple). Data are averages ±1 SE (n = 4 plots for survival, in each plot n = 5 juveniles initially and thereon the number varied depending on the number of surviving juveniles). Superimposed circles represent results of SNK test for survival data at Feb. 09 in La Vela. Size of natural (non-transplanted, n = 80) recruits at each site is also presented (red). As all plots at Due Sorelle were destroyed by the last monitoring point due to storms, thus size data for this site are presented only until Oct 08.

Juveniles of *C. barbata* transplanted onto more stable habitats had higher survival rates than those transplanted back into their original location on loose boulders, but the final survival rates varied between study sites and treatments (Fig. 9a–b, Site x Treatment interaction close to significant, *F*_3,24_ = 2.78, P = 0.06). By October 2008, controls supported the least surviving juveniles at both sites (Fig. 9a–b). At La Vela the trend persisted over time, and by February 2009 there where no surviving juveniles in control plots compared to 20–70% surviving juveniles in the other treatments (significant effect of Treatment, *F*_3,12_ = 6.75, P<0.01). Conversely at Due Sorelle, no juveniles survived the storm of December 2008. Survival in the other treatments differed between the two study sites, without any clear or consistent trend (Fig. 9a–b), e.g., at La Vela survival was greatest on vertical substrata, while at Due Sorelle survival was greatest on horizontal substrata. Interestingly, there were no differences in relation to presence or absence of an adult canopy at neither sites. In general surviving juveniles showed rates of growth comparable among treatments and similar to natural rates of growth measured for un-manipulated recruits, suggesting that the transplantation did not impair the juveniles' growth.

## Discussion

We have documented the severe decline of forests of canopy-forming Fucoid algae along the coasts of Monte Conero. This decline is part of a regional [34], [43] and global trend [15], [16], [17] in vegetated marine habitats. Forests of Fucoids used to be diverse and abundant in the Adriatic Sea [44]. Nowadays losses have been documented at numerous locations, particularly along the Italian coastline ([34], [40], [43], [45], S. Fraschetti pers. comm. to LA, the present study), where the scattered and fragmented canopies of *C. barbata* we have described are among the last few remaining.

The decline of the *Cystoseira* forests was rather sudden, with >70% of the canopies lost during 2002–2005. The proximate trigger appears to be related to combined acute disturbance from beach nourishments (in the form of gravel and cobbles) and severe storms which were frequent during this period. However, available historical data suggest that the loss was probably facilitated by long-term natural and human-induced changes in the biotic and abiotic conditions in the system, including a decline in the number of species composing the forest (from at least 8 known in 1941 to only 2 in the 1990s), along with increased bedrock instability. Both these factors could have contributed to the erosion of the long-term resilience of the system.

Studies on terrestrial and other marine systems suggest that diverse assemblages may have higher chances for recovery in response to perturbations than depleted ones. The importance of individual species in driving ecosystem responses has not been well explored in canopy forming marine systems [46], and the mechanisms underlying the historical widespread declines of *Cystoseira* species are notoriously difficult to disentangle [22]. It is well known, however, that healthy canopies exert a strong control on the physical and biological environment, for example by sweeping away detrimental sediments or inhibiting potential competitors such as algal turfs [21], [47], [48], thus triggering positive feedbacks that facilitate self recruitment and maintenance [23]. Recent experimental work in other canopy systems [49] also reports that canopies formed by different species may differently affect both the physical and biological environments, which could be related to variations in morphology and habitat architecture of the species. Understanding how the progressive degradation and decline in species richness alter the structure, function and resilience of marine canopy forests should be a critical focus of future research in these systems.

The instability of rocky bedrocks, due to hydrodynamic forces (tides, waves, currents, seasonal storms), can abrade and destroy canopy macroalgae on rock surfaces significantly affecting their distributions [22], [50], [51], [52]. However, the impacts of human-induced physical modification of the habitat as possible drivers for both decline and prolonged lack of natural recovery of algal forests have been so far overlooked. Humans can directly or indirectly modify the characteristics of bottom habitats. In the study area, for example, the natural instability of the rocky bottom has been historically enhanced by rock mining, and more recently by extensive additions of gravel and quarry stones for nourishments of tourist cobble beaches. The analysis of natural recruitment patterns showed that recovery of canopies was virtually precluded in unstable rocky habitats, due to severe damage and mortality from boulder displacement and overturning during severe storms. Moreover, transplantation experiments demonstrated that substratum stabilization significantly increased the survival of juveniles. These results, combined with data on the dynamic of canopy loss and wave regime in the region, strongly suggest that historical natural- and human-induced increased bedrock instability may have reduced the resilience of canopy forests against climate-driven threats in the form of severe storms, and transforming occasional acute disturbances into chronic stress, which makes natural recovery slow or even implausible. This not only suggests that hydrodynamic processes and substratum stability can play an important role in regulating the loss of canopy algae, but also demonstrates that the synergistic interactions between multiple stressors can exacerbate nonlinear responses of ecosystems to human impacts and limit their adaptive capacity [6], [11], [53]. Bottom instability is likely to increase in the future, due to expected greater coastal erosion, and increased storminess [42], [54]. Management actions focused on reducing the risk of further loss of canopy forests are therefore particularly urgent.

Our study reinforces the notion that the loss of marine algal forests represents a shift to an alternative state dominated by species of less structural complexity, such as turf-forming, filamentous or other ephemeral seaweeds, mussels or urchin barrens [11], [21], [24], [25], where continued changes have deviated the system from its natural state so much so that recovery is slowed or comes to a standstill. The habitat shift (i.e. the hysteresis effect) was evident from clearing and transplantation experiments, which demonstrated that increased substratum stability is essential but not sufficient to return to a canopy dominated state, and that for recovery to occur removal of the new dominant space occupiers is also required. Recovery is always the result of interactions between species (dominance, inhibition or facilitation) and extrinsic factors (e.g. colonist supply and environmental setting) [5], [28], and there are circumstances where such interactions can feedback negatively limiting recovery for decades [17], [29]. Informing managers about whether specific ecosystems are likely to exhibit alternative stable states and regime shifts, and the potential for reversing undesired shifts, is the first fundamental step to stimulate and guide the identification of policy initiatives aimed at promoting ecosystem resilience.

Consistent recruitment failure may be a key explanation for persistent habitat fragmentation and loss in many systems. Lack of surrounding adult canopies did not seem to impair the potential recovery of the system, suggesting that in these systems recovery could be actively enhanced even following severe depletions. The latter is valid as long as an adequate supply of propagules is maintained and human-mediated conditions that prevent the recovery of the systems are reversed. This would include: limiting further beach nourishment in the area; actively stabilizing the habitat, for example by removing or fixing to place small-medium sized boulders; and facilitating recruitment on stable substrata during the reproductive season, through extensive clearing of opportunistic benthic components, or by limiting growth of opportunistic forms through an improved management of water quality as suggested by Goeman et al [25]. The transplantation experiment also suggested that active translocation of recruits from loose substratum onto secure and stable zones of the habitat, can be effective in facilitating system recovery. This approach might be particularly efficient in cases where forests are highly fragmented and isolated, or spore supply is limited, and translocation of recruits could expand the area over which canopies can persist.

A great deal of time and money is put into the challenging problem of restoring natural habitats world-wide. Such efforts are critical for preservation of biodiversity, as well as of the critical socioeconomic resources provided by many natural habitats [3], [55]. Our results suggest that the loss of marine habitats in human-dominated landscapes could be mitigated with appropriate consideration and management of incremental changes in relevant habitat characteristics and of attributes facilitating the recovery in these systems. They also provide an empirical demonstration of the potential benefits from a more integrated management approach to resilience, where the focus is on reducing risks of major ecosystem changes rather than on individual environmental stressors [5]. This conceptual shift is particularly important in face of the escalating degradative ecological change of our coasts and oceans [16]. We need to provide better protection to marine habitats, as it is becoming increasingly clear that mere restoration of environmental conditions preceding a loss, if possible, may be insufficient for ecosystem restoration, and is scarcely cost-effective.
